# Hypoxia preconditioned bone marrow-derived mesenchymal stromal/stem cells enhance myoblast fusion and skeletal muscle regeneration

**DOI:** 10.1186/s13287-021-02530-3

**Published:** 2021-08-09

**Authors:** Karolina Archacka, Iwona Grabowska, Bartosz Mierzejewski, Joanna Graffstein, Alicja Górzyńska, Marta Krawczyk, Anna M. Różycka, Ilona Kalaszczyńska, Gabriela Muras, Władysława Stremińska, Katarzyna Jańczyk-Ilach, Piotr Walczak, Mirosław Janowski, Maria A. Ciemerych, Edyta Brzoska

**Affiliations:** 1 Department of Cytology, Institute of Developmental Biology and Biomedical Sciences, Faculty of Biology, University of Warsaw, Miecznikowa 1 St, 02-096 Warsaw, Poland; 2 Department of Histology and Embryology, Medical University of Warsaw, 02-004 Warsaw, Poland; 3 Laboratory for Cell Research and Application, Medical University of Warsaw, 02-097 Warsaw, Poland; 4 Department of Pathophysiology, Faculty of Medical Sciences, University of Warmia and Mazury, Warszawska 30 St, 10-082 Olsztyn, Poland; 5 Russell H. Morgan Department of Radiology and Radiological Science, Division of MR Research, the Johns Hopkins University School of Medicine, Baltimore, MD 21205 USA; 6 Center for Advanced Imaging Research, Department of Diagnostic Radiology and Nuclear Medicine, University of Maryland, Baltimore, MD 21201 USA; 7 NeuroRepair Department, Mossakowski Medical Research Centre, Polish Academy of Sciences, Pawinskiego 5 St, 02-106 Warsaw, Poland

**Keywords:** BM-MSC, Fusion, Hypoxia, Normoxia, Migration, Myogenic differentiation

## Abstract

**Background:**

The skeletal muscle reconstruction occurs thanks to unipotent stem cells, i.e., satellite cells. The satellite cells remain quiescent and localized between myofiber sarcolemma and basal lamina. They are activated in response to muscle injury, proliferate, differentiate into myoblasts, and recreate myofibers. The stem and progenitor cells support skeletal muscle regeneration, which could be disturbed by extensive damage, sarcopenia, cachexia, or genetic diseases like dystrophy. Many lines of evidence showed that the level of oxygen regulates the course of cell proliferation and differentiation.

**Methods:**

In the present study, we analyzed hypoxia impact on human and pig bone marrow-derived mesenchymal stromal cell (MSC) and mouse myoblast proliferation, differentiation, and fusion. Moreover, the influence of the transplantation of human bone marrow-derived MSCs cultured under hypoxic conditions on skeletal muscle regeneration was studied.

**Results:**

We showed that bone marrow-derived MSCs increased *VEGF* expression and improved myogenesis under hypoxic conditions in vitro. Transplantation of hypoxia preconditioned bone marrow-derived MSCs into injured muscles resulted in the improved cell engraftment and formation of new vessels.

**Conclusions:**

We suggested that SDF-1 and VEGF secreted by hypoxia preconditioned bone marrow-derived MSCs played an essential role in cell engraftment and angiogenesis. Importantly, hypoxia preconditioned bone marrow-derived MSCs more efficiently engrafted injured muscles; however, they did not undergo myogenic differentiation.

**Supplementary Information:**

The online version contains supplementary material available at 10.1186/s13287-021-02530-3.

## Introduction

Skeletal muscle regeneration is a complex process that allows restoration of skeletal muscle homeostasis lost due to the injury, such as intensive exercise, surgical procedures, and diseases. Skeletal muscle regeneration covers two distinct phases. The first one includes tissue degeneration, accompanied by inflammation, necrosis of damaged myofibers, and their phagocytosis by immune cells. The second one is regeneration, leading to new myofiber formation followed by their maturation, tissue reinnervation, and finally skeletal muscle functional recovery [1]. Muscle necrosis occurs when myofibers’ integrity is severely disrupted what involves increased sarcolemma permeability, organelle dysfunction, and loss of myofiber architecture. Necrotic myofibers release many cytokines, growth factors, and chemoattractants. These signals activate tissue-resident and circulating inflammatory cells [2, 3]. Neutrophils are the first to infiltrate the site of injury. These cells phagocytize damaged myofibers and release numerous factors which induce migration of local monocytes and their differentiation into macrophages [4–6]. Two days after injury, macrophages become the predominant cell population present within damaged tissue [3, 7, 8]. They can be divided into two distinct subpopulations—M1, also considered as pro-inflammatory macrophages, characterized by the presence of CD68, responsible for phagocytosis of necrotic tissue, and releasing pro-inflammatory factors, such as tumor necrosis factor α (TNF-α), interleukin-1β (IL-1β), IL6, IL12, as well as nitric oxide (NO) and reactive oxygen species (ROS) [9–11]. The second described population is M2, also called anti-inflammatory macrophages, characterized by the presence of CD163, releasing factors, like tumor growth factor β (TGF-β), IL4, IL10, or IL13, and for supporting myoblast differentiation, angiogenesis, and extracellular matrix (ECM) deposition [12, 13]. The next phase of skeletal muscle repair covers myofiber regeneration which is possible due to satellite cells (SCs)—skeletal muscle-specific stem cells, characterized by a PAX7 transcription factor. These cells are tightly connected to the myofibers and located between basal lamina and sarcolemma. In healthy muscles, SCs remain quiescent, but after injury, they become activated, reenter the cell cycle, start to proliferate, differentiate into myoblasts which further fuse to form myotubes. Finally, myotubes’ maturation leads to new functional myofibers’ formation [5, 14–16].

The myogenic differentiation of SCs is regulated by sequentially expressed transcription factors, called myogenic regulatory factors (MRFs). MRF family consists of MYF5, MYOD, myogenin, and MRF4 [17, 18]. Quiescent SCs are characterized by the presence of paired box transcription factor 7 (PAX7). PAX7 and MYF5 are present in proliferating SCs and myoblasts. PAX7 regulates the expression of MYF5 and MYOD, while MYF5 presence enhances the expression of MYOD. MYOD is a critical factor of myogenic differentiation. It facilitates the transition from myoblast proliferation to the myocyte differentiation stage by inducing the myogenin, p21, and p57 cell cycle inhibitor expression [19, 20]. Further, MYOD and myogenin trigger the expression of other genes essential for muscle cell function, such as MRF4, myosin heavy and light chains, muscle creatine kinase, or troponin [21]. The expression of myogenin and MRF4 is accompanied by the downregulation of PAX7, MYF5, and MYOD. Some cells do not undergo differentiation but remain PAX7 positive, downregulate MYOD, and restore the SC population necessary for the next rounds of muscle regeneration [22–24]. Those that differentiated fuse to each other to result in the formation of multinucleated myotubes and then myofibers [25, 26]. Alternatively, differentiated myocytes can fuse with already existing myofibers during the regeneration of slightly damaged skeletal muscles [27]. Finally, newly formed myotubes and myofibers undergo maturation to become fully functional. During maturation, myofibers grow, myofibers’ proper contractility is restored, and neuromuscular junctions are formed [28, 29].

In skeletal muscle diseases, sarcopenia, or cachexia, skeletal muscle regeneration is disturbed. Many populations of stem and progenitor cells are studied for potential therapeutic use. Two main strategies to support skeletal muscle regeneration are considered. First, the transplanted cells could participate in myofiber reconstruction; second, transplanted cells’ secreted factors could support regeneration. One of the studied cells are bone marrow-derived stromal cells, also known as bone marrow-derived “mesenchymal” stem/stromal cells (bone marrow-derived MSCs). However, it should be noted that these cells do not present naïve myogenic potential [30]. Bone marrow-derived MSCs are a heterogeneous population [31], typically isolated from bone marrow on the basis of their ability to adhere to the culture plate’s surface. It was proven that bone marrow-derived MSCs contain a population of cells that fulfill the rigorous criteria of stem cells [32]. This subpopulation of bone marrow-derived MSCs present long-term expansion without phenotypic change, self-renewal probed during in vivo serial transplantations, and multipotency examined by in vivo differentiation assay at the single-cell level [32–36]. CD146 appeared to be a handy marker to select and isolate stem cell subpopulations from bone marrow-derived MSCs [33]. Human CD146 + bone marrow-derived MSCs were shown to be able to self-renew, differentiate into bone and bone marrow, as well as to support organization of endothelial cells into functional blood vessels, and differentiate into chondrocytes and adipocytes [33, 37].

As we mentioned above, bone marrow-derived MSCs do not present naïve myogenic potential [33]. These cells do not fuse in the absence of myoblasts and rarely fuse with myoblasts in co-cultures [33, 38–41]. However, bone marrow-derived MSCs could follow myogenic differentiation as the result of reprogramming induced by 5-azacytidine treatment, overexpression of Notch intracellular domain (NICD), paired box transcription factor 3 (Pax3), or constitutively active β-catenin, or as a result of 3D co-culture with myofibers [42–47]. It was also documented that bone marrow-derived MSCs could support skeletal muscle regeneration; however, these cells rarely participate in new myofiber formation [38, 47–52].

In the current study, we focused on the hypoxia effect on bone marrow-derived MSC and myoblast co-cultures. We also followed if cultured under hypoxic condition bone marrow-derived MSCs could more efficiently support skeletal muscle regeneration. The level of oxygen is an essential factor regulating gene transcription and cell fate. The level of O_2_ during in vitro culture under hypoxic conditions (1–3%) is much more similar to the level present in the physiological bone marrow-derived MSC niche in the bone marrow (2–7%) than that observed under standard in vitro culture conditions. Accordingly, it was previously shown that bone marrow-derived MSCs cultured under hypoxic conditions induced their proliferation, migration, elevated colony-forming unit capabilities, increased ECM deposition, osteogenic and adipogenic potential, and angiogenic factor expression [53–59].

Moreover, preconditioning of bone marrow-derived MSC under hypoxia increased their ability to engraft injured tissues after transplantation. In the subacute murine limb ischemia model, hypoxia preconditioned bone marrow-derived MSCs injected into skeletal muscles engrafted this tissue more efficiently, induced neoangiogenesis, and improved blood flow [57]. Similar results were observed after transplantation of hypoxia preconditioned bone marrow-derived MSCs to other ischemic tissues, including heart, brain, lung, and liver [58–62]. We hypothesized that hypoxic preconditioning impacts the human bone marrow-derived MSC secretome. As a result, these cells could more efficiently engraft injured skeletal muscle, support myoblast fusion, and skeletal muscle regeneration. To follow this problem, we choose to investigate human and pig bone marrow-derived MSCs. We selected cells of two species as we previously showed that as far as MSCs are concerned, the cells’ origin may determine their reaction to the same factors [63]. Moreover, pig serves as a valuable model in preclinical research. We analyzed human and pig bone marrow-derived MSCs in vitro, co-cultured with mouse myoblasts, and in vivo after their transplantation to mouse injured skeletal muscles.

## Materials and methods

### Primary myoblast, C2C12, and MSC culture under normoxic and hypoxic conditions

Four different cell types were used during experiments. Human bone marrow-derived mesenchymal stromal cells (hMSCs) were obtained from Lonza (Lonza PT-2501). Fetal pig bone marrow-derived mesenchymal stromal cells (pMSCs) were kindly provided by dr. Joanna Wojtkiewicz from University of Warmia and Mazury in Olsztyn. pMSCs were isolated from transgenic pigs, which constitutively expressed green fluorescent protein (GFP). Both types of MSCs were cultured in Dulbecco’s modified Eagle’s medium (DMEM; ThermoFisher Scientific) containing glucose 4.5 g/l supplemented with 15% inactivated fetal bovine serum (FBSin; ThermoFisher Scientific) and 0,1% gentamycin solution (Sigma-Aldrich), further referred to as MSC medium (MSCmed). C2C12 cells (Sigma-Aldrich) were cultured in DMEM containing glucose 4.5 g/l, supplemented with 10% FBSin and 1% penicillin–streptomycin solution (ThermoFisher Scientific), further referred to as C2C12 medium (C2C12med). Mouse primary myoblasts (mPM) were isolated from *tibialis anterior* (TA), *soleus*, *extensor digitorum longus (*EDL), and *flexor digitorum brevis* (FDB) muscles of 2–3-month-old C57/BL6 male mice using Bischoff and Rosenblatt method [64, 65]. Briefly, muscles were isolated from tendon to tendon, digested in 0.2% collagenase type I (Sigma-Aldrich) solution in DMEM. Then, single myofibers, devoid of any contaminating cells, were collected in suspension, passed through a 21G syringe needle, and filtered through a 40 μm strainer. Primary myoblasts were cultured in DMEM containing glucose 1 g/l, supplemented with 10% horse serum (HS; ThermoFisher Scientific), 20% FBSin, 0.5% chicken embryo extract (CEE; ThermoFisher Scientific), and 1% penicillin–streptomycin solution, further referred to as PM medium (PMmed). All cell types were cultured in normoxia (37 °C, 21% O_2_, 5% CO_2_) or hypoxia (37 °C, 5% O_2_, 5% CO_2_).

### Migration assay—scratch assay

Migration of pMSCs or hMSCs cultured either under hypoxia or normoxia was analyzed using scratch wound healing assay [66]. Briefly, cells were cultured to obtain 90–100% confluence. Next, the cells were scratched from the plate using a plastic tip to create the “wound.” The wound healing manifested by the ability of the cells to refill the created gap was observed. After 3.5 h, 8 h, and 24 h cells were fixed with cold methanol and stained using Giemsa–May–Grünwald method. The pictures were taken, and the area of the scratch was calculated using GIMP 2.

### Myoblast and bone marrow-derived MSC co-culture, fusion index, and hybrid myotube analysis

Co-cultures were obtained by seeding mPM in a 1:1 ratio with either pMSCs or hMSCs. Cells were cultured under normoxic or hypoxic conditions in MSCmed or PMmed for 5–7 days. C2C12 myoblasts (3 × 10^4^ or 6 × 10^4^) were cultured in the absence of hMSCs or pMSCs or co-cultured with hMSCs or pMSCs in 3:2.5; 3:5; 3:7.5 ratio. Cells were cultured under normoxic or hypoxic conditions in C2C12med for 5–7 days. Further, cells were fixed, and fusion index or proportion of hybrid myotubes were estimated.

Fusion index of C2C12 or mPM cultured alone or in co-cultures with either pMSCs or hMSCs was calculated. Briefly, differentiated cells were fixed in cold methanol and stained according to the Giemsa–May–Grünwald method. Images from 4 fields of view were collected, and nuclei number was counted. Fusion index was calculated as a percentage of nuclei present in myotubes compared to all visible cell nuclei.

Myotubes formed by either C2C12 or PM co-cultured with either pMSCs or hMSCs were visualized using skeletal myosin’s immunolocalization. The participation of hMSCs in myotube formation was evaluated by visualization of human nuclei. pMSC contribution in hybrid myotube formation was verified by the presence of GFP within myotubes.

### Co-culture of human bone marrow-derived MSC and myoblasts without direct contact

The 1 × 10^4^ of hMSCs were cultured in cell culture inserts in the presence of 1 × 10^4^of PMs in the lower dish, under normoxic or hypoxic conditions, in MSCmed or PMmed, for 5–7 days. Such co-culture conditions secured that hMSCs had no physical contact with myoblasts. Then, hMSCs and PMs were counted and separately collected, and RNA was isolated for further analysis.

### Muscle injury and cell transplantation

Local Ethics Committee No. 1 in Warsaw, Poland, approved all procedures involving animals—permission number: 669/2018. To induce skeletal muscle injury, 2–3-month-old NOD SCID mice (Janvier Labs) were anesthetized, and their *gastrocnemius* muscles were injected with 50 μl of 10 mM cardiotoxin (L8102, Latoxan). Further, 24 h after cardiotoxin treatment 0.5 × 10^6^ of hMSCs cultured in normoxia or hypoxia (for 48 h) and suspended in 20 μl of phosphate-buffered saline (PBS) were injected into damaged *gastrocnemius* muscle. In contrast, the contralateral muscle was injected with 0.9% NaCl solution (such saline-treated muscles served as a control). After 14 days of regeneration, mice were killed, and muscles were isolated and analyzed.

### Immunocytochemistry and immunohistochemistry

Selected antigens were immunolocalized in in vitro cultured cells as well as in muscle cross sections. In vitro cell cultures were fixed with 3% PFA, washed with PBS, and stored in 4 °C. Dissected skeletal muscles were frozen in isopentane, cooled down with liquid nitrogen, transferred to -80 °C, and cut into 10 μm sections using cryomicrotome (Microm HM, Thermo Fisher Scientific). Cryosections were fixed with 3% paraformaldehyde, washed with PBS, and stored in 4 °C. Further fixed cells or cryosections were permeabilized with 0.1% Triton X-100 (Sigma-Aldrich) in PBS and incubated with 0.25% glycine (Sigma-Aldrich) in PBS. Non-specific binding of antibodies was blocked with 3% bovine serum albumin (BSA, Sigma-Aldrich) in PBS. Then cells or cryosections were incubated with primary antibodies diluted 1:100 in 3% BSA in PBS overnight followed by incubation with appropriate secondary antibodies conjugated with fluorochromes, diluted 1:200 in 1.5% BSA in PBS for 2 h in room temperature. Next, samples were washed with PBS, and cell nuclei were visualized with Hoechst 33,342 diluted 1:500 in 3% BSA in PBS. Finally, specimens were mounted with Fluorescent Mounting Medium (Dako Cytomation). Samples were analyzed using Axiovert 100 M LSM 510 (Zeiss) and ZEN software. The following primary antibodies were used: rabbit anti-mouse skeletal myosin (M7523; Sigma-Aldrich), mouse anti-human nuclear antigen (ab191181; Abcam), rabbit anti-laminin (L9393; Sigma-Aldrich). The following secondary antibodies were used: donkey anti-mouse conjugated with AlexaFluor 488 (A21202, ThermoFisher Scientific) or AlexaFluor 594 (A21203, ThermoFisher Scientific), goat anti-rabbit conjugated with AlexaFluor 488 (A11008, ThermoFisher Scientific), or donkey anti-rabbit conjugated with AlexaFluor 594 (A21207, ThermoFisher Scientific).

### Muscle histology

Dissected skeletal muscles were frozen in isopentane, cooled down with liquid nitrogen, transferred to − 80 °C, and cut into 10 μm sections using cryomicrotome (Microm HM, Thermo Fisher Scientific). Cryosections were fixed with 3% PFA, washed with PBS, and stored in 4 °C. Samples were hydrated in PBS, incubated in Harris hematoxylin solution (Sigma-Aldrich), and washed in distilled water. Then, fixed sections were incubated in eosin Y solution (Sigma-Aldrich) and washed in distilled water. Specimens were mounted with UltraMount (Dako Cytomation) and analyzed using inverted light microscope Eclipse TE200 (Nikon) and ImageJ software (NIH).

### Gene expression analysis

Total RNA was isolated from muscles, C2C12, mPMs, pMSCs, and hMSCs cultured alone or in co-cultures, using High Pure Isolation Kit (Roche) and from dissected muscles using mirVana™ miRNA Isolation Kit (Thermo Fischer Scientific) and purified with Turbo DNA-free Kit (Thermo Fischer Scientific), according to the manufacturers’ protocols. cDNA was obtained in reverse transcription reaction performed using RevertAid First Strand cDNA Synthesis Kit (ThermoFisher Scientific) according to manufacturer’s protocol. The conditions of reverse transcription were as follows: 25 °C for 10 min, 42 °C for 60 min, 85 °C for 5 min. Next, mRNA levels were examined using quantitative real-time PCR analysis (qPCR) with TaqMan assays (ThermoFisher Scientific) for the following genes: human: *CD9* (Hs00233521_m1)*, ADAM9* (Hs00177638_m1)*, CSPG4* (Hs00361541_g1), *PDFGRB* (Hs01019589_m1), *VWF* (Hs00169795_m1), *KDR* (Hs00911700_m1), *CDH15* (Hs00170504_m1), *MYOD1* (Hs02330075_g1)*, MYF5* (Hs00929416_g1)*, MYOG* (Hs01072232_m1), *MCAM* (Hs00174838_m1), *VCAM1* (Hs01003372_m1), *NES* (Hs04187831_g1), *CXCL12* (Hs03676656_mH), *VEGFA* (Hs05484830_s1), *WNT4* (Hs01573505_m1), *FAP* (Hs00990791_m1); pig: *SGCA* (Ss03821424_s1)*, ACTA1* (Ss04245853_m1), *DES* (Ss03378045_u1), *MYOG* (Ss03379073_u1); mouse*: Adam9* (Mm01218460_m1)*, Cd9* (Mm00514255_g1), *Cdh15* (Mm00483191_m1), *Ncam1* (Mm01149710_m1), *Vcam1* (Mm01320970_m1), *Pax7* (Mm01354484_m1), *Myf5* (Mm00435125_m1), *Myod1* (Mm00440387_m1), *Myog* (Mm00440387_m1), *Cxcl12* (Mm004485552_m1), *Vegfa* (Mm00437304_m1), *Vwf* (Mm00550376_m1), *Kdr* (Mm01222419_m1)*. HPRT/Hprt* (Hs99999909_m1, Ss03382484_u1, Mm03024075_m1) was used as a reference gene for in vitro studies and *actin (ACTB*; Hs030233943_g1 and Mm01205647_g1) was used as reference gene for in vivo studies*.* All reactions were performed in duplicates. qPCR was performed with the TaqMan Gene Expression Master Mix (ThermoFisher Scientific) using LightCycler 480 (Roche) according to manufacturer’s instruction. The conditions of qPCR were as follows: preincubation 2 min., 50 °C; preincubation 10 min., 95 °C; amplification (40 cycles) 15 s., 95 °C, and 1 min., 60 °C. Expression levels were calculated with 2^−(ΔCT)^ formula.

### Statistical analysis

At least three independent biological experiments were shown as mean with standard deviations with GraphPad Prism 7. The results were analyzed in GraphPad Prism 7 with the one-way ANOVA test and Bonferroni multicomparison test or Student t test (Fig. 5). The results were compared to cells cultured in MSCmed under normoxic conditions.

## Results

### The proliferation, migration, and fusion of human and pig bone marrow-derived MSCs, mouse primary myoblasts, and C2C12 under normoxic and hypoxic conditions

**Fig. 1:**
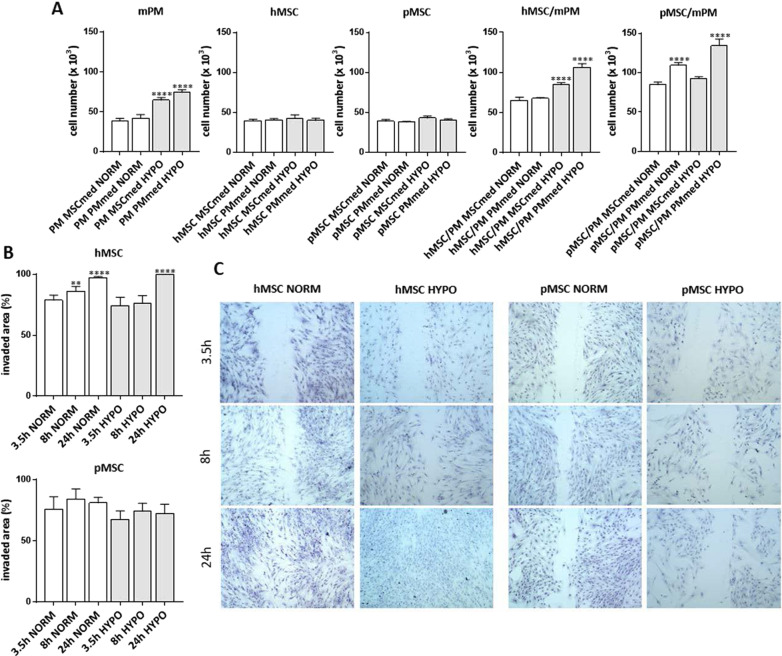
Cell proliferation and migration under normoxic and hypoxic conditions. **A **The number of mouse primary myoblasts (mPM), human bone marrow-derived mesenchymal stromal cells (hMSC), pig bone marrow-derived mesenchymal stromal cells (pMSC), cells in co-cultures of hMSC and mPM, and cells in co-cultures of pMSC and mPM, cultured in two types of medium: MSCmed and PMmed, under normoxic (NORM) or hypoxic (HYPO) conditions. **B **The invaded area measured in scratch wound healing assay of hMSC and pMSC cultured in MSCmed under normoxic and hypoxic conditions. **C** The scratch wound healing assay of hMSC and pMSC cultured in MSCmed under normoxic and hypoxic conditions. *P* value: *< 0.05; **< 0.01; ***< 0.001; ****< 0.0001

First, we analyzed the proliferation of human bone marrow-derived mesenchymal stromal cells (hMSC), fetal pig bone marrow-derived mesenchymal stromal cells (pMSC), as well as mouse primary myoblasts (mPM), which we used to set up the co-culture experiments (Fig. 1A). Next, we performed similar analyzes of hMSC or pMSC co-cultured with mPM or mouse C2C12 myoblasts. The two types of myoblasts were analyzed because of differences between primary cultures and cell lines. All these experiments were carried out either under normoxic or hypoxic conditions. Cells and co-cultures were analyzed after 5–7 days of culture in the following media types: MSCmed and either C2C12med or PMmed. MSCmed and C2C12med allowed studying the cells cultured under proliferating conditions and PMmed under differentiating conditions. Analysis of mPMs showed that their number was significantly higher in cultures carried under hypoxic conditions, regardless of the medium used. Neither hypoxia nor the type of medium influenced the number of hMSCs or pMSCs. Co-culture of mPMs with either hMSCs or pMSCs, conducted under hypoxic conditions, also increased overall cell number (Fig. 1A). The hMSCs and pMSCs were able to migrate. The changes in hMSC migration were significant after 8 h and 24 h in normoxia, as well after 24 h in hypoxia, comparing to cells cultured for 3.5 h in normoxia. However, no significant change in hMSC or pMSC migration was noticed comparing normoxic and hypoxic conditions (Fig. 1B.). The differences in migration between cells cultured in normoxia and hypoxia and analyzed after 3.5 h or 24 h were not statistically significant.

**Fig. 2:**
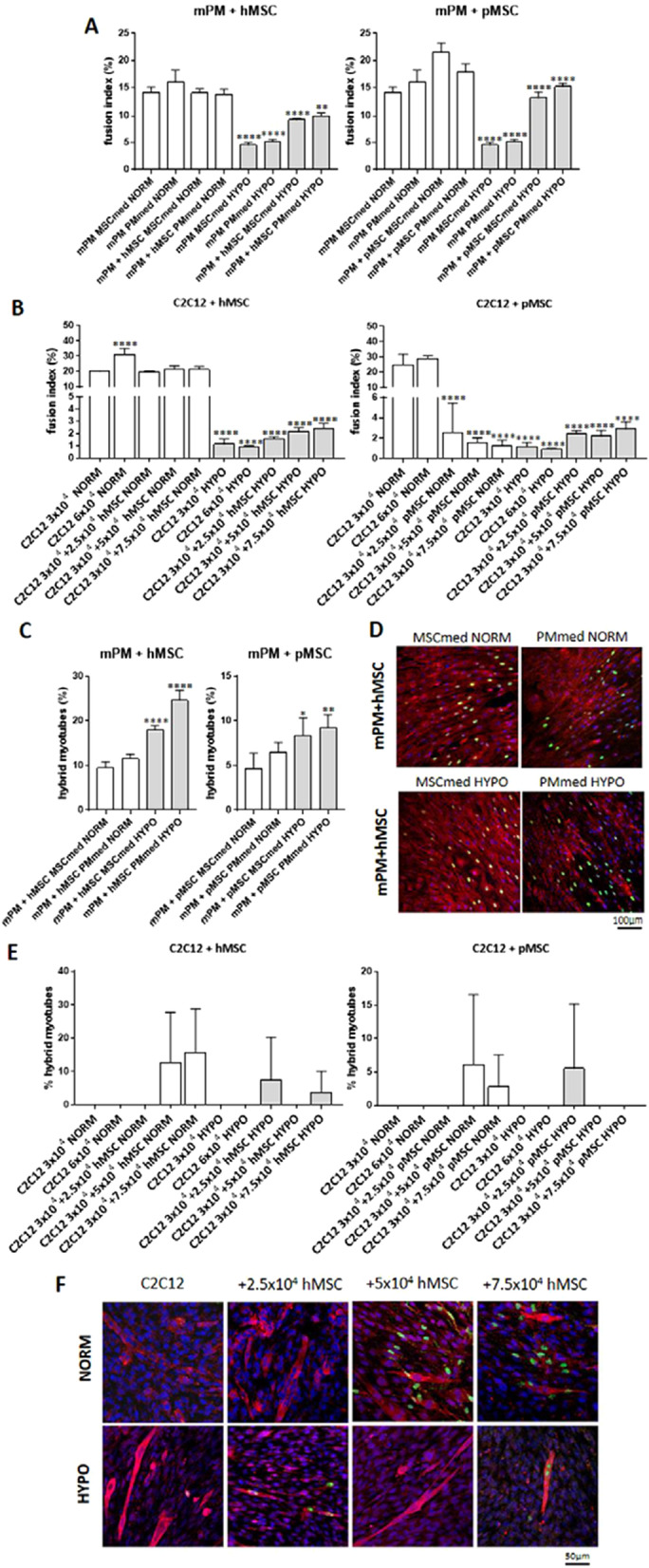
Fusion index and hybrid myotube presence in either human bone marrow-derived mesenchymal stromal cells (hMSC) or pig bone marrow-derived mesenchymal stem cells (pMSC) co-culture with myoblasts: mouse primary myoblasts (mPM) or C2C12 myoblasts. **A** Fusion index of mPM and hMSC or pMSC co-cultured in MSCmed or PMmed under normoxic (NORM) or hypoxic (HYPO) conditions. **B** Fusion index of C2C12 myoblasts and hMSC or pMSC co-cultured in 3:2.5; 3:5; 3:7.5 ratio, in C2C12med, under normoxic (NORM) or hypoxic (HYPO) conditions. **C** Percentage of hybrid myotubes in mPM and hMSC or pMSC co-cultures in MSCmed or PMmed under normoxic (NORM) or hypoxic (HYPO) conditions. **D** Immunolocalization of hybrid myotubes in mPM and hMSC co-cultures, red—skeletal myosin, blue—cell nuclei, green human cell nuclei. **E** Percentage of hybrid myotubes in C2C12 and hMSC or pMSC co-cultures in C2C12med under normoxic (NORM) or hypoxic (HYPO) conditions. **F** Immunolocalization of hybrid myotubes in C2C12 and hMSC co-cultures, red—skeletal myosin, blue—cell nuclei, green—human cell nuclei. *P* value: *< 0.05; **< 0.01; ***< 0.001; ****< 0.0001

Second, we compared the fusion index and proportion of hybrid myotubes formed due to the fusion between either hMSCs or pMSCs with either mPM or C2C12 myoblasts. Depending on the experimental set, the co-cultures were conducted in MSCmed, C2C12med, or PMmed, in each case under normoxic or hypoxic conditions (Fig. 2). First, we analyzed the fusion index of mPM (Fig. 2A) or C2C12 myoblasts (Fig. 2B). We noticed that hypoxic conditions significantly decreased myoblast fusion. Next, fusion was considerably higher when mPM and C2C12 were cultured under hypoxic conditions in the presence of either hMSCs or pMSCs. In the case of co-cultures conducted under normoxic, the presence of hMSCs did not impact the fusion index of mPM nor C2C12. Interestingly, pMSCs had a negative impact on C2C12 myoblast fusion when cultured under normoxic conditions. The proportion of hybrid myotubes formed by mPM with either hMSCs or pMSCs significantly increased under hypoxic conditions (Fig. 2C, D). The fusion of hMSCs or pMSCs and C2C12 differed depending on the culture medium, and hypoxic conditions did not increase hybrid myotubes’ formation (Fig. 2D, F).

### The changes in expression of selected markers in human and pig bone marrow-derived MSCs and mouse primary myoblasts under normoxic and hypoxic conditions

**Fig. 3:**
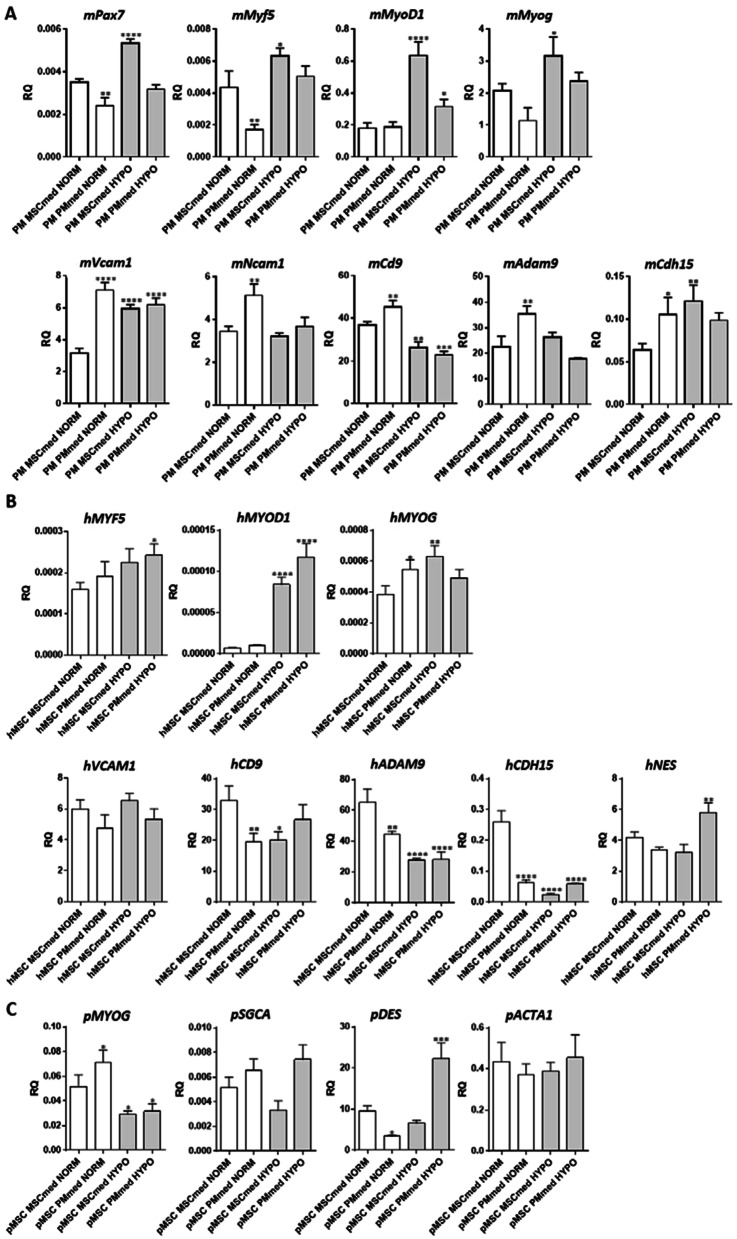
Expression of selected markers in cell cultures. **A** Level of transcripts encoding PAX7, MYF5, MYOD1, myogenin (MYOG), VCAM1, NCAM1, CD9, ADAM9, m-cadherin (CDH15) in mouse primary myoblasts (mPM) cultured in PMmed or MSCsmed under normoxic (NORM) or hypoxic (HYPO) conditions. **B** Level of transcripts encoding MYF5, MYOD1, myogenin (MYOG), VCAM1, CD9, ADAM9, m-cadherin (CDH15), nestin (NES) in human bone marrow-derived mesenchymal stromal cells (hMSC) cultured in PMmed or MSCsmed under normoxic (NORM) or hypoxic (HYPO) conditions. **C** Level of transcripts encoding myogenin (MYOG), α-sarcoglycan (SGCA), desmin (DES), and actin α1 (ACTA1) in pig bone marrow-derived mesenchymal stem cells (pMSC) cultured in PMmed or MSCsmed under normoxic (NORM) or hypoxic (HYPO) conditions. *P* value: *< 0.05; **< 0.01; ***< 0.001; ****< 0.0001

To follow the changes in myogenic differentiation of cells cultured under normoxic and hypoxic conditions in two different types of media (MSCmed and PMmed), the expression of transcript encoding PAX7, myogenic regulatory factors (MRFs), cytoskeletal proteins, and adhesion proteins was examined in mouse, human, and pig cells (Fig. 3). The expression of mouse *Pax7, Myf5, Myod1,* and myogenin (*Myog*) significantly increased in mPM cultured under hypoxic conditions, regardless of the culture medium used (Fig. 3A). The higher level of mRNAs encoding Pax7 and MRFs in cells cultured under hypoxic conditions corresponded to myoblasts’ higher proliferation. It is well known that *Pax7, Myod1,* and *Myf5* are expressed in activated satellite cells, and *Myod1* and *Myf5* in proliferating cells [17]. The expression of MRFs in hMSCs and pMSCs was barely detectable (Fig. 3B, C). Human *MYF5*, *MYOD1, and MYOG* level increased in hMSCs cultured under hypoxic conditions but was still very low (Fig. 3B).

The level of mRNAs encoding adhesion proteins such as VCAM, NCAM, CD9, ADAM9, and m-cadherin (*cdh15 or CDH15*) which are engaged in cell–cell adhesion and myoblast fusion [67–74] depended on the cell type and culture conditions used, i.e., medium and oxygen level (Fig. 3A, B). Their expression was significantly higher in mPM cultured in PMmed than in MSCmed under normoxia. Moreover, we observed a significant decrease in *Cd9* expression in mPM cultured under hypoxic conditions, regardless of medium type (Fig. 3A). It corresponded to less efficient fusion of myoblasts observed under hypoxic conditions (Fig. 2A). Analysis of hMSCs documented a significant decrease in *ADAM9, CDH15*, and *CD9* expression levels when cells were cultured under hypoxic conditions, regardless of the medium used. In the case of pMSCs, the level of α-sarcoglycan (*SGCA*) and desmin (*DES*) transcripts, i.e., encoding proteins characteristic for muscle cells [75, 76], changed dependently on culture conditions, being the highest in cells cultured in PMmed under hypoxic conditions (Fig. 3C).

**Fig. 4:**
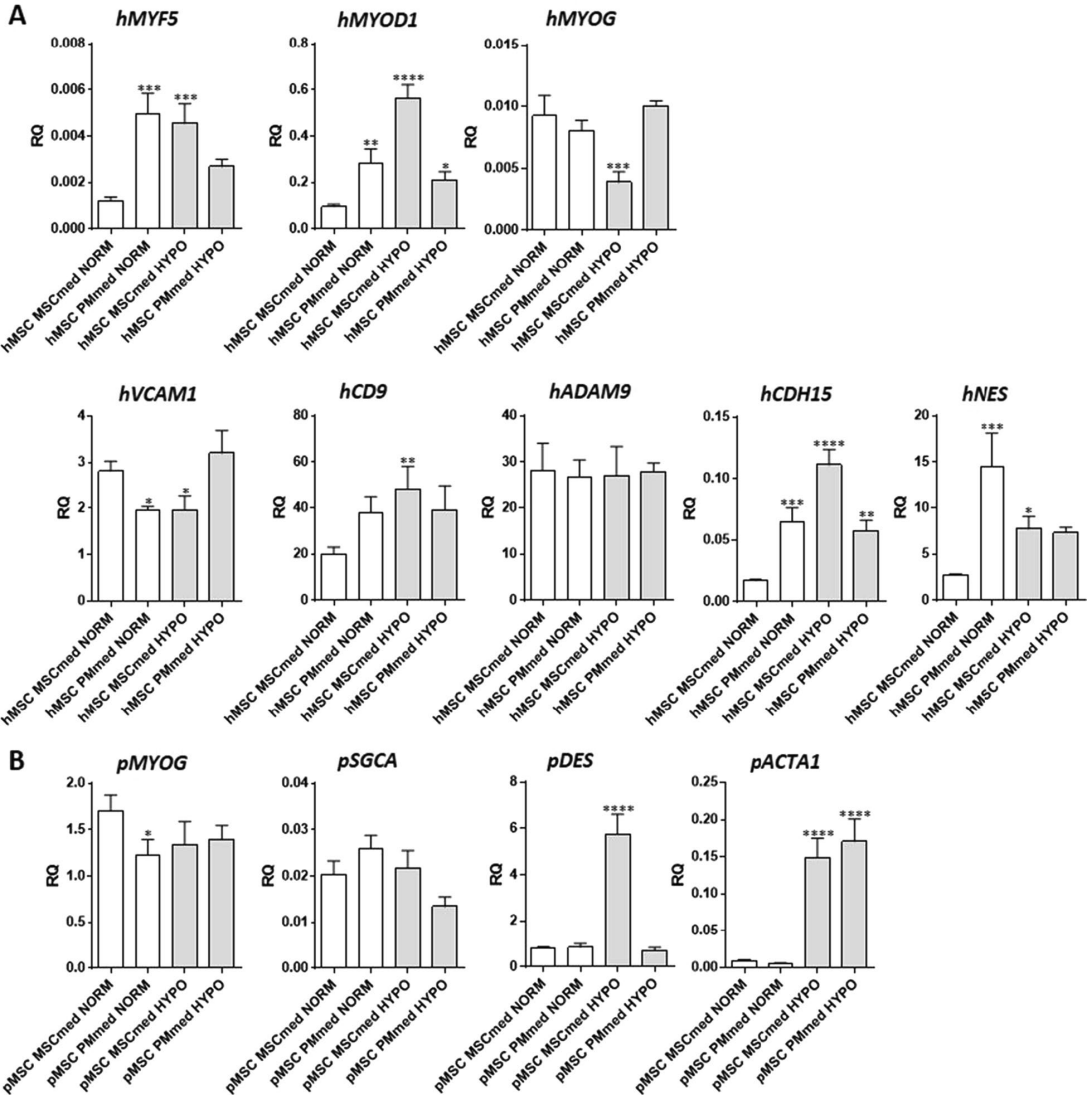
Expression of selected markers in cell co-cultures. **A** Level of human transcripts encoding MYF5, MYOD1, myogenin (MYOG), VCAM1, CD9, ADAM9, m-cadherin (CDH15), nestin (NES) in co-cultures of mouse primary myoblasts (mPM) and human bone marrow-derived mesenchymal stromal cells (hMSC) in PMmed or MSCsmed under normoxic (NORM) or hypoxic (HYPO) conditions. **B** Level of pig transcripts encoding myogenin (MYOG) SGCA, desmin (DES), and actin α1 (ACTA1) in mPM and pig bone marrow-derived mesenchymal stem cells (pMSC) co-cultured in PMmed or MSCsmed under normoxic (NORM) or hypoxic (HYPO) conditions. *P* value: *< 0.05; **< 0.01; ***< 0.001; ****< 0.0001

Co-culture of hMSCs or pMSCs cells with mPMs or C2C12 myoblasts were analyzed to establish the changes in the expression of transcripts encoding MRFs, cytoskeletal, and adhesion proteins in human and pig cells cultured in the presence of myoblasts, under both normoxic or hypoxic conditions (Fig. 4). Hypoxia increased hybrid myotube formation in co-cultures between either hMSCs or pMSCs and mPMs, in comparison with co-cultures conducted under normoxia. The level of human MRFs was significantly higher in hMSCs co-cultured with mPM than hMSCs cultured alone under all culture conditions tested (Figs. 3B and 4A). Thus, the presence of myoblasts impacted the MRF expression in hMSCs. Moreover, we detected a higher expression level of human *MYF5* and *MYOD1* in co-cultures with mouse myoblasts carried in PMmed under normoxic conditions and in both types of the medium under hypoxic conditions (Fig. 4A). Also, the level of transcripts encoding human *CD9* and *CDH15* significantly increased in cells cultured under hypoxic conditions. Hypoxia and myoblasts’ presence did not alter the *MYOG* expression in pMSCs, but the high level of *DES* (desmin) was noticed in cells cultured in MSCmed (Fig. 4B).

To follow the changes in proliferation, differentiation, and expression of selected transcripts in co-cultures carried out without physical contact, we seeded mPMs in dishes to which inserts with hMSCs were placed. Such experimental approach allowed us to check how the secretom of both types of cells mutually influence their proliferation, differentiation, and gene expression, under normoxic and hypoxic conditions. In such experimental setting mPM number increased under hypoxic conditions, regardless of the medium used (Additional file 1: Figure S1). The number of hMSCs did not change (Additional file 1: Figure S1). Thus, we concluded that hypoxia impacts mPM but not hMSC proliferation. Moreover, the levels of human and mouse *MYOD1, MYOG, CD9, CDH15*, and transcripts encoding secreted proteins engaged in cell mobilization and differentiation, such as *CXCL12* (SDF-1*), VEGF,* and *VWF,* were analysed (Additional file 1: Figure S1). We showed that the expression of MRFs in hMSCs was barely detectable (Additional file 1: Figure S1). Human *MYOD1*, and *MYOG* expression level increased under hypoxic conditions but was still very low (Additional file 1: Figure S1). Thus, the presence of myoblasts and physical contact between analyzed cells impacted the MRF expression in hMSCs. Similary, the trend of changes in adhesive protein transcripts, i.e., *CD9* and *CDH15* corresponded to hMSC culture but not hMSC and mPM co-culture. Under hypoxic conditions, the level of human *VEGF* in hMSCs increased; however, the changes were not statistically significant. We did not observe statisticaly significant changes in expression of selected transcripts in mPM (Additional file 1: Figure S1). However, the level of mouse *Cd9* significantly decreased in cells cultured in PMmed under hypoxic conditions (Additional file 1: Figure S1).

### The transplantation of human bone marrow-derived MSCs cultured under normoxic and hypoxic conditions into mouse injured skeletal muscles

**Fig. 5:**
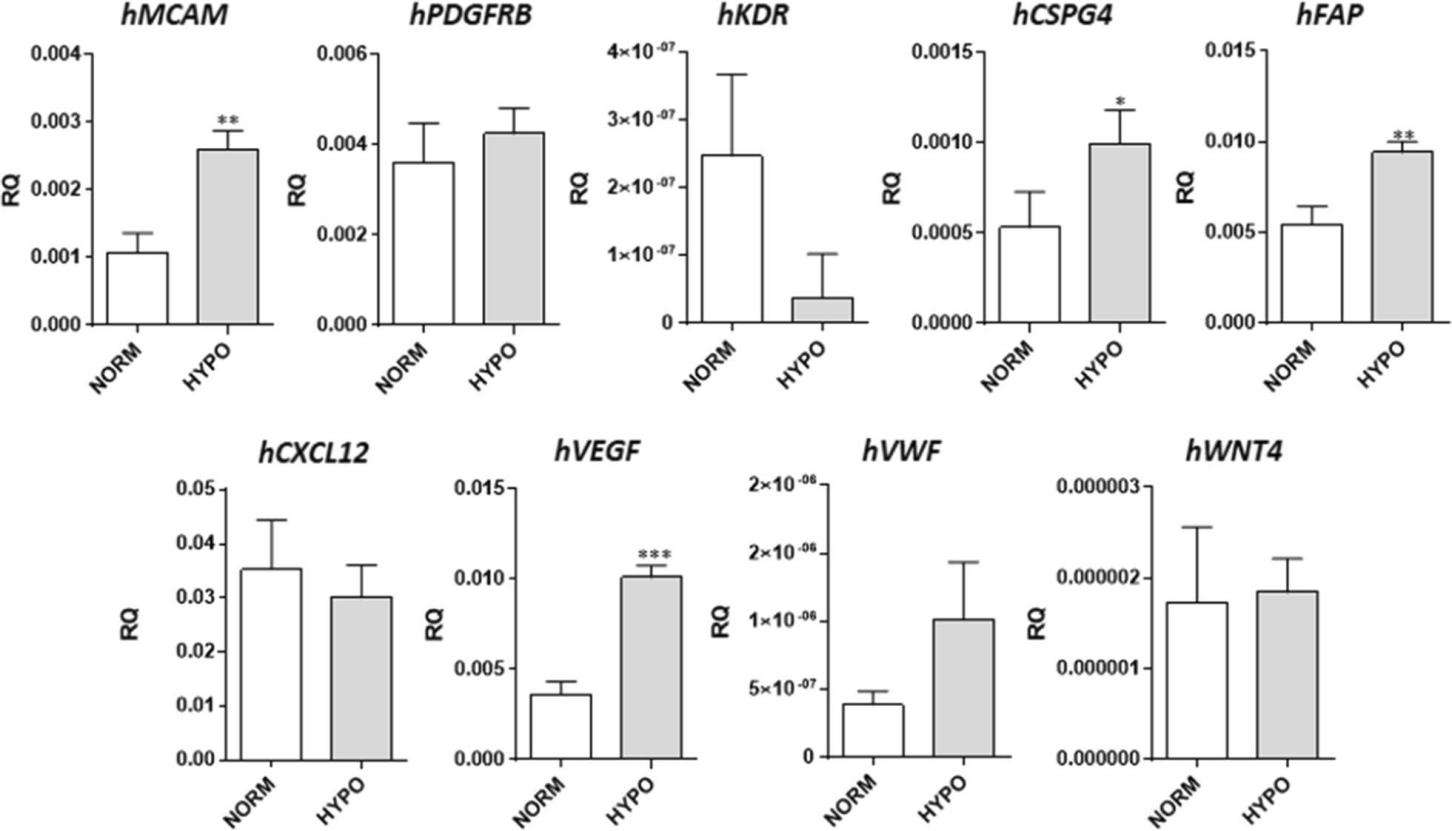
Level of selected marker expression in human bone marrow-derived mesenchymal stromal cells (hMSC) cultured in MSCsmed under normoxic (NORM) or hypoxic (HYPO) conditions. *P* value: *< 0.05; **< 0.01; ***< 0.001; ****< 0.0001

As we demonstrated, human and pig bone marrow-derived MSCs influenced myoblasts’ proliferation and fusion when cultured under hypoxic conditions. Since  none significant differences between human and pig cells’ impact on mouse myoblasts were found, we decided to inject human bone marrow-derived MSCs into injured mouse muscle. Before transplantation, we examined the expression of human progenitor cell (*MCAM/CD146*), pericyte (*PDGFRb, NG2*), endothelial (*VEGFR*), and fibroblast (*FAP*) markers in hMSCs cultured in MSCmed under normoxic and hypoxic conditions (Fig. 5). CD146 was shown to be a marker of a subpopulation of human bone marrow-derived stem cells [88, 89]. We noticed that the expression of *MCAM/CD146* and *NG2* (*CSPG4*) increased under hypoxic conditions. However, the expression of *FAP* also increased. The level of *PDGFRb* and *KDR *(*VEGFR*) did not change significantly under hypoxic conditions. Then we analyzed the level of transcripts of secreted proteins engaged in cell mobilization and differentiation, such as SDF-1 (CXCL12), VEGF, VWF, and WNT4. We found that the level of *VEGF* increased in hMSCs cultured under hypoxic conditions.

**Fig. 6:**
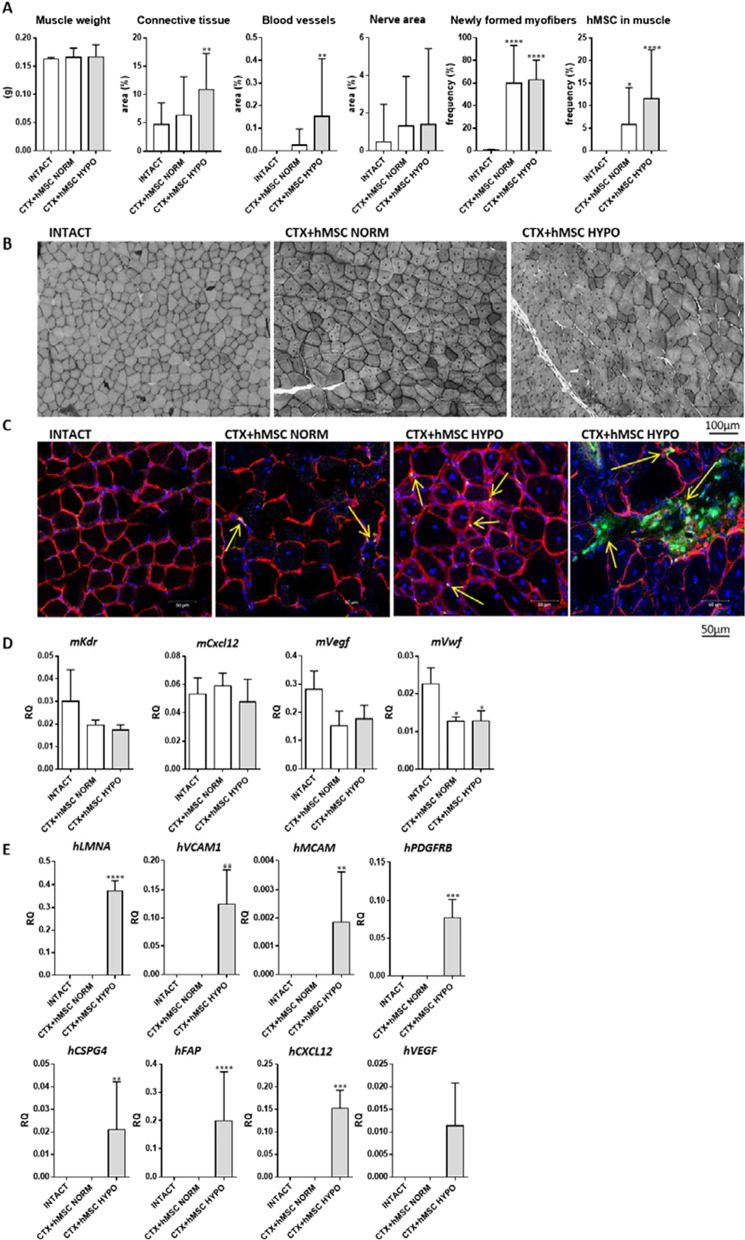
Skeletal muscle regeneration after human bone marrow mesenchymal stromal cell (hMSC) transplantation. The hMSCs were cultured under normoxic (NORM) or hypoxic (HYPO) conditions and injected into cardiotoxin (CTX) injured muscles. **A** Skeletal muscle weight, the area of connective tissue, blood vessels, nerves, and frequency of new myofibers in intact muscles or muscles injured (CTX) and transplanted with hMSC cultured either under normoxic (NORM) or hypoxic (HYPO) conditions. **B** Histology of intact muscles or in muscles injured (CTX) and transplanted with hMSC cultured under normoxic (NORM) or hypoxic (HYPO) conditions. **C** Localization of transplanted cells (blue—nuclei, red—laminin, green—human nuclei) in intact muscles or muscles injured (CTX) and transplanted with hMSC cultured under normoxic (NORM) or hypoxic (HYPO) conditions. **D** Expression of selected mouse transcripts in intact muscles or muscles injured (CTX) and transplanted with hMSC cultured under normoxic (NORM) or hypoxic (HYPO) conditions. **E** Expression of selected human transcripts in intact muscles or muscles injured (CTX) and transplanted with hMSC cultured under normoxic (NORM) or hypoxic (HYPO) conditions. *P* value: *< 0.05; **< 0.01; ***< 0.001; ****< 0.0001

Finally, hMSC cultured under normoxic conditions and hypoxia preconditioned hMSCs were transplanted into ctx injured skeletal muscle of SCID mice (Fig. 6). The muscle mass, number of newly formed myofibers, and nerve area did not differ between muscles that received hMSCs cultured under either normoxic or hypoxic conditions (Fig. 6A, B). After 14 days of regeneration, the presence of newly formed myofibers with centrally located nuclei was noticed, regardless of the type of culture (Fig. 6B). However, the area of connective tissue was higher in muscles injected with hMSCs cultured under hypoxic conditions, as compared to control muscles (Fig. 6A, B). Importantly, the area of blood vessels was higher after hypoxia preconditioned hMSC transplantation. Moreover, a higher number of human cells was detected in mouse muscles injected with hypoxia preconditioned hMSCs (Fig. 6A, C). These cells were found between myofibers (Fig. 6C). Then, we analyzed the level of mouse and human transcripts after cell transplantation. The level of mouse *Vwf* was lower in injured muscles than in intact muscle; however, it did not differ between muscles transplanted with hMSC cultured under either standard or hypoxic conditions (Fig. 6D). Notably, only in mouse muscles injected with hypoxia preconditioned hMSCs the human transcripts such as laminin, *VCAM, MCAM, PDGFRb, CSPG4* (NG2), *FAP, CXCL12* (SDF-1), and *VEGF* were found (Fig. 6E). Besides, the *WNT*, *MYH3, MYF5, MYOD1,* and *MYOG* transcripts were not detected. We concluded that hypoxia preconditioned hMSCs efficiently engrafted injured muscle but did not follow myogenic differentiation based on obtained results.

## Discussion

Under physiological conditions, the oxygen level in healthy resting human skeletal muscles equates from 3 to 4% (25–34 mmHg) [77], dropping to 7.5 mmHg during intense exercise [78]. In resting mouse muscle, the oxygenation reaches approximately 50 mmHg O_2_ [78]. All these values are significantly lower than oxygen pressure under standard cell culture, amounting circa 142 mmHg (i.e., approx. 20% O_2_). For this reason, cell cultures performed under hypoxic conditions (oxygen pressure below 50 mmHg, 2–6% O_2_) are considered more physiological, as shown for bone marrow and muscle cells [78, 79]. The level of oxygen influences many cellular processes regulating the activity of O_2_-consuming enzymes such as cytochrome c oxidase or prolyl hydroxylase, as well as influencing on ROS formation [78–81]. One of the essential proteins responsible for cell reaction to O_2_ level changes is hypoxia-inducible factor 1 (HIF-1), which regulates hypoxia responsive genes [82]. The O_2_ level impacts inter alia, cell proliferation, migration, differentiation, viability, protein synthesis, and secretion [78].

In our study, increase in the primary myoblast proliferation was observed in cultures conducted under hypoxic conditions, what stays in agreement with other studies showing the higher proliferation of rat, human, and mouse primary myoblasts cultured in hypoxia (2–6% O_2_) [83–86]. The C2C12 proliferation also increased in co-cultures with mouse bone marrow-derived MSCs in a VEGF-dependent manner [87]. Importantly, we observed the increased *VEGF* expression in human bone marrow-derived MSCs cultured under hypoxic conditions. However, human and pig bone marrow-derived MSC proliferation or migration was comparable, regardless of culture conditions. On the other hand, mouse bone marrow-derived MSCs migrated more efficiently, in transmembrane migration assay, in response to conditioned medium under hypoxic conditions [56]. Human bone marrow-derived MSCs cultured under hypoxic conditions increased their migration rates and HGF receptor expression, i.e., c-Met [88]. Thus, the influence of hypoxia on bone marrow-derived MSC migration abilities depends on many variables. Interestingly, hypoxia was shown to affect rat bone marrow-derived MSC response to chemokines, inflammatory cytokines, and growth factors [89].

In the current study, we documented that mouse primary myoblasts and C2C12 myoblasts fused less efficiently under hypoxic conditions. Other studies showed that the impaired fusion of C2C12 myoblasts cultured under hypoxic conditions was also associated with myotube atrophy and a lower number of nuclei per myotube [90]. Also, mouse primary and H-2 K myoblast differentiation were less efficient under such conditions [91]. Moreover, most of the studies described the inhibition of myogenic differentiation under hypoxia [79].

Quiescent satellite cells reside in hypoxic niche what induces expression of HIF2A, which maintains their quiescence and self-renewal, and blocks differentiation [77]. We noticed that the expression of *Pax7* and MRFs, i.e., *MyoD, Myf5*, and *Myog*, increased in myoblasts under hypoxic conditions. The higher level of *Pax7* and MRF expression results in elevated proliferation of myoblasts in hypoxia. Moreover, hypoxia was shown to activate Notch1, leading to a reduction in miR1 and miR206 expression and PAX7 upregulation [92]. Lower level of fusion-engaged tetraspanin CD9 observed by us could result in impaired fusion of analyzed myoblasts [70, 71, 93, 94].

We noticed that fusion of myoblasts changed in the presence or absence of bone marrow-derived MSCs. As long as the bone marrow-derived MSCs and myoblasts were co-cultured under standard conditions, the fusion did not change significantly. Under hypoxic conditions, the presence of human or pig bone marrow-derived MSCs significantly improved myoblast fusion, i.e., enhanced myoblast differentiation and the formation of hybrid myotubes. To identify the mechanism determining such phenomenon, we examined the changes in human or pig MRF, adhesion proteins, cytoskeletal proteins, and other myoblast markers, such as dystrophin or sarcoglycan expression, in bone marrow-derived MSCs and co-cultures of bone marrow-derived MSCs and myoblasts. The level of human or pig MRF mRNAs was very low or undetectable. It, however, increased under hypoxic conditions, but only in human bone marrow-derived MSCs. The expression level of mRNAs encoding adhesion proteins engaged in cell fusion varied depending on cell culture medium and conditions. Moreover, changes observed in MRF and adhesion protein expression were dependent on physical contact bettwen the hMSCs and mPMs. Then, we found that under hypoxic conditions, expression of *VEGF* significantly increased in human bone marrow-derived MSCs. It was also documented that mouse bone marrow-derived MSCs overexpressed *Vegf* and secreted more VEGF under hypoxic conditions [55, 56]. Moreover, both mouse primary myoblasts and C2C12 myoblasts expressed *Vegf* and its receptors [95]. VEGF enhanced C2C12 myoblast migration, differentiation, and prevented apoptosis, what resulted in myotubes hypertrophy [87, 95, 96]. Thus, VEGF could be responsible for improved myoblast proliferation and differentiation observed by us under hypoxic conditions.

Finally, we transplanted hypoxia preconditioned human bone marrow-derived MSCs to injured skeletal muscles of SCID mice to study their influence on tissue regeneration. Bone marrow-derived MSCs cultured under hypoxia more efficiently engrafted the muscle. However, they were found only between myofibers. We were able to detect expression of human *LMNA*, *VCAM, NG2, CD146, PDGFR,* and *FAP* in muscles after hypoxia preconditioned bone marrow-derived MSC transplantation with more effective engraftment. However, we did not notice the presence of myofibers formed by human bone marrow-derived MSCs, and the expression of human *MYF5, MYOD, MYOG,* and *MYH3* was not detected. Thus, we concluded that hypoxia preconditioned bone marrow-derived MSCs more efficiently engrafted injured muscles but did not follow myogenic differentiation. Notably, the human *SDF-1* and *VEGF* transcripts were present in mouse muscle after hypoxia preconditioned bone marrow-derived MSC transplantation, and these factors could impact skeletal muscle regeneration. We suggested that VEGF, which is upregulated in hypoxia preconditioned bone marrow-derived MSCs, could play a vital role in cell engraftment after transplantation. Verma and coworkers showed that tissue-resident satellite cells expressed VEGF, which recruited endothelial cells [97]. In this way, satellite cells induced capillary formation in their niche. We suggested that improved expression of VEGF in hypoxia preconditioned bone marrow-derived MSCs could induce vessel formation and support cell engraftment. A higher number of vessels was found in muscles after hypoxia preconditioned bone marrow-derived MSC transplantation. Reconstruction of vessel network is essential for muscle reconstruction, and the reduction of skeletal muscle network was described in dystrophic, ALS, or denervated muscles [98]. Acute muscle damage led to disruption in the microvasculature, hypoxia, and activation of HIF-1α signaling—the main factor of hypoxic response [99]. One of the HIF-1 target genes is *VEGF*, i.e., a well-described factor triggering angiogenesis also in skeletal muscles [99]. VEGF and angiogenesis improved skeletal muscle regeneration and chronic skeletal muscle diseases [96, 99–101]. Similarly, SDF-1 expression increased in injured muscles, presented a proangiogenic effect, and mobilized stem cells to injured muscles [102–106]. The restoration of blood flow and vascular formation was also enhanced after intra-arterial injection of hypoxia preconditioned mouse bone marrow-derived MSCs to mice with hind limb ischemia [88]. Hypoxia preconditioned mouse bone marrow-derived MSC transplantation increased WNT4 expression in skeletal muscles, and WNT4 was shown to induce bone marrow-derived MSC proliferation and migration as well as endothelial cell migration and myoblast differentiation [88]. Based on all the above-mentioned results, we concluded that SDF-1 and VEGF secreted by hypoxia preconditioned bone marrow-derived MSCs increased new vessel formation during skeletal muscle reconstruction.

## Conclusions

The hypoxia induced proliferation of myoblasts but delayed their differentiation, decreased CD9 and increased PAX7 and MRFs transcripts expression. The bone marrow-derived MSCs significantly improved myoblast proliferation and differentiation in co-cultures under hypoxic conditions in vitro in a VEGF-dependent manner. Moreover, bone marrow-derived MSCs more frequently fused in vitro with myoblasts under hypoxic conditions. Hypoxia preconditioning of bone marrow-derived MSCs increased the level of VEGF expression. Such cells more efficiently engrafted injured muscles in vivo but did not follow myogenic differentiation. Their transplantation into injured muscles increased, however, muscle mass and new vessels’ formation in a SDF-1- and VEGF-dependent manner.

## Supplementary Information

**Additional file 1: Figure S1**. Proliferation and expression of selected human and mouse markers (*MyoD1, Myog, Cd9, Cdh15, Vegf, Sdf-1, Vwf)* in cell co-culture of human bone marrow-derived mesenchymal stromal cells (hMSC) and mouse primary myoblasts (PM) without direct/physical contact. The hMSCs were cultured in cell culture inserts in the presence of PM in the lower dish in PMmed or MSCsmed under normoxic (NORM) or hypoxic (HYPO) conditions.

## Data Availability

The datasets used and/or analysed during the current study are available from the corresponding author on reasonable request.

## Abbreviations

ACTA1: Actin α1, skeletal muscle
ACTB: Actin β
ADAM9: A Disintegrin And Metalloproteinase Domain 9
Akt: Protein kinase B
BSA: Bovine serum albumin
CD: Cluster of differentiation
CDH15: M-cadherin
CEE: Chicken embryo extract
CSPG4/NG2: Neural-glial antigen 2
CXCL12/SDF1: C-X-C chemokine 12/stromal-derived factor 1
DAMP: Damage-associated molecular pattern
DES: Desmin
DMEM: Dulbecco’s modified Eagle’s medium
ECM: Extracellular matrix
EDL: Extensor digitorum longus
FAP: Fibroblast activation protein
FBSin: Fetal bovine serum inactivated
FDB: Flexor digitorum brevis
FGF: Fibroblast growth factor
FOXO: Forkhead box O
GFP: Green fluorescent protein
HGF: Hepatocyte growth factor
HGFR/c-Met: Hepatocyte growth factor receptor
HIF: Hypoxic-inducible factor
hMSC: Human mesenchymal stem/stromal cell
HPRT: Hypoxanthine phosphoribosyltransferase
HS: Horse serum
IGF-1: Insulin-like growth factor 1
IL: Interleukin
KDR/VEGFR: Vascular endothelial growth factor receptor
MAPK: Mitogen-activated protein kinase
MCAM/CD146: Melanoma cell adhesion molecule
MMP: Metalloproteinase
mPM: Mouse primary myoblasts
MRF: Myogenic regulatory factor
MRF4: Myogenic regulatory factor 4
MSC: Mesenchymal stem/stromal cell
MSCmed: Mesenchymal stromal cells medium
mTOR: Mammalian target of rapamycin
MYF5: Myogenic factor 5
MYH3: Myosin heavy chain 3, embryonic
MYOD: Myoblast determination protein
MYOG: Myogenin
NADPH: Nicotinamide adenine dinucleotide phosphate (reduced)
NCAM1: Neural cell adhesion molecule 1
NES: Nestin
NFATc: Nucelar factor of activated T-cells, cytoplasmic
NF-κB: Nuclear factor kappa B
NICD: Notch intracellular domain
NO: Nitric oxide
Pax3: Paired box transcription factor 3
Pax7: Paired box transcription factor 7
PBS: Phosphate-buffered saline
PDFGRB: Platelet-derived growth factor receptor β
PFA: Paraformaldehyde
PI3K: Phosphoinositide 3-kinase
PMmed: Primary myoblasts medium
pMSC: Pig mesenchymal stem/stromal cell
ROS: Reactive oxygen species
SC: Satellite cell
SCID: Severe combined immunodeficiency
SGCA: α Sarcoglycan
STAT3: Signal transducer and activator of transcription 3
TA: Tibialis anterior
TGF-β: Tumor growth factor β
TNF-α: Tumor necrosis factor α
TRPC: Transient receptor potential cation channel
VCAM: Vascular cell adhesion molecule
VEGF: Vascular endothelial growth factor
VWF: Von Willebrand factor
WNT: Wingless/integrated
