# Supplementary figures and images for: Hypoxia preconditioned bone marrow-derived mesenchymal stromal/stem cells enhance myoblast fusion and skeletal muscle regeneration
Source: Stem Cell Res Ther. 2021 Aug 9;12:448. doi: 10.1186/s13287-021-02530-3 (PMC8351116; doi:10.1186/s13287-021-02530-3)

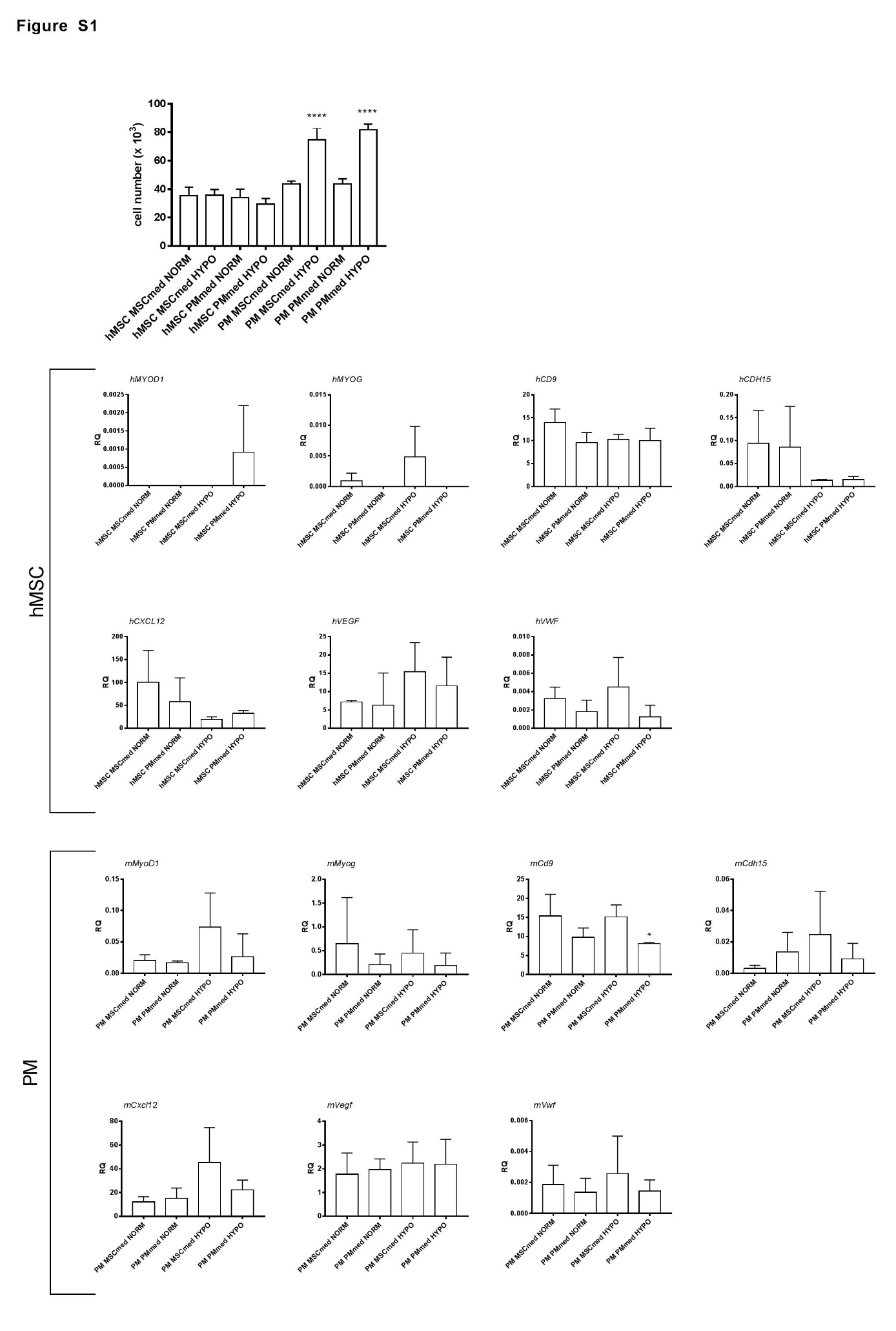

Supplement: Supplementary file 1 — Additional file 1: Figure S1. Proliferation and expression of selected human and mouse markers (MyoD1, Myog, Cd9, Cdh15, Vegf, Sdf-1, Vwf) in cell co-culture of human bone marrow-derived mesenchymal stromal cells (hMSC) and mouse primary myoblasts (PM) without direct/physical contact. The hMSCs were cultured in cell culture inserts in the presence of PM in the lower dish in PMmed or MSCsmed under normoxic (NORM) or hypoxic (HYPO) conditions. [file 13287_2021_2530_MOESM1_ESM.docx]
